# Acute Urinary Retention Secondary to Urethral Lithiasis in a 4-Year-Old Boy: How We Managed This Rare Case

**DOI:** 10.1055/a-2663-1933

**Published:** 2025-08-05

**Authors:** Thibault Planchamp, Pierre Estournes, Adrien Boileau, Solène Joseph, Mathilde Piraprez, Florian Laclergerie, Luana Carfagna, Olivier Abbo

**Affiliations:** 1 Department of Pediatric Surgery, Hôpital des Enfants, Toulouse, Occitanie, France; 2 Department of Urology, Hôpital de Rangueil, Toulouse, Occitanie, France

**Keywords:** urolithiasis, urethral calculi, pediatric urology, laser lithotripsy, endoscopy

## Abstract

Urethral stone impaction (USI) is an extremely rare cause of acute urinary retention (AUR) in pediatric urology. Few case reports are available, and no consensus guidelines currently exist for managing this condition. We describe our management of such a case and provide a review of the relevant literature. A 4-year-old boy with no prior urological history presented to our emergency department with abdominal pain lasting 8 days. An ultrasound performed 5 days earlier identified a 7-mm stone at the ureterovesical junction. Symptomatic treatment with paracetamol and non-steroidal anti-inflammatory drugs was initiated. However, dysuria, painful penile swelling, and AUR developed 7 days after the onset of pain. A CT scan revealed bilateral hydronephrosis, urinary retention, and a 9-mm stone (980 Hounsfield Units) that had migrated to the proximal anterior urethra. Under general anesthesia, a suprapubic puncture removed 400 mL of urine for analysis. A 7.5-Fr cystoscope was used to identify an impacted stone at the base of the penile urethra. In situ lithotripsy was performed using a holmium laser to fragment the stone in the urethra. The fragments were then pushed into the bladder for complete disintegration. Intravesical fragments were subsequently removed with a Dormia basket. No urethral wounds were observed, and a 10-Fr catheter was placed. Recovery was uneventful, with catheter removal and spontaneous voiding on postoperative day 1. At the 3-month follow-up, the patient exhibited normal voiding and uroflowmetry. AUR secondary to USI is rare and lacks standardized management protocols in pediatric urology. Management of USI should be tailored to the size and location of the calculus, as well as the presence of any associated urethral pathology, with a preference for minimally invasive endoscopic surgery whenever possible. If necessary, urethral in situ laser lithotripsy appears to be a safe and effective treatment option to consider.

## Introduction

Urolithiasis in children is an increasingly common condition, with a growing incidence of 1 to 5% in advanced countries and 5 to 15% in developing countries. 1

Obstruction may occur anywhere along the urinary tract, from the kidney to the urethral meatus. Guidelines are available for the management of renal, ureteric, or bladder lithiasis. 2

However, with an incidence of 0.3% in adults, urethral stone impaction (USI) is 20 times rarer in children. 3 It can lead to both acute and chronic complications and can be challenging to manage.

Only a few case reports are available for pediatric patients, and no consensus guidelines currently exist for the management of this condition. Moreover, in situ urethral laser lithotripsy remains underreported in pediatric urology, underscoring the need for new case reports with long-term follow-up to provide valuable insights into its feasibility and outcomes.

Here, we present our first case of acute urinary retention (AUR) secondary to USI in a young boy, successfully treated with intraurethral holmium laser lithotripsy.

## Case Presentation

**Video 1** Step-by-step illustration of the surgical procedure: Manual localization of the stone in the penile urethra; suprapubic aspiration for bladder drainage; in situ urethral laser lithotripsy using a holmium laser set at 0.8 J and 8 Hz.


A 4-year-old boy (weight: 17.3 kg; height: 106 cm; body mass index: 15.4 kg/m ^2^ ), with a past medical history of severe epilepsy currently treated with carbamazepine, clobazam, and lamotrigine, was admitted to our emergency department (ED) for abdominal pain lasting 8 days and had progressed to dysuria, painful penile swelling, and AUR since this morning.

An ultrasound performed 5 days earlier identified a 7-mm stone at the ureterovesical junction. Symptomatic treatment with paracetamol and non-steroidal anti-inflammatory drugs was then initiated by the general practitioner.

At the ED, vital signs were stable, and there was no fever. However, the patient was in severe pain, requiring continuous intravenous nalbuphine.

**Fig. 1:**
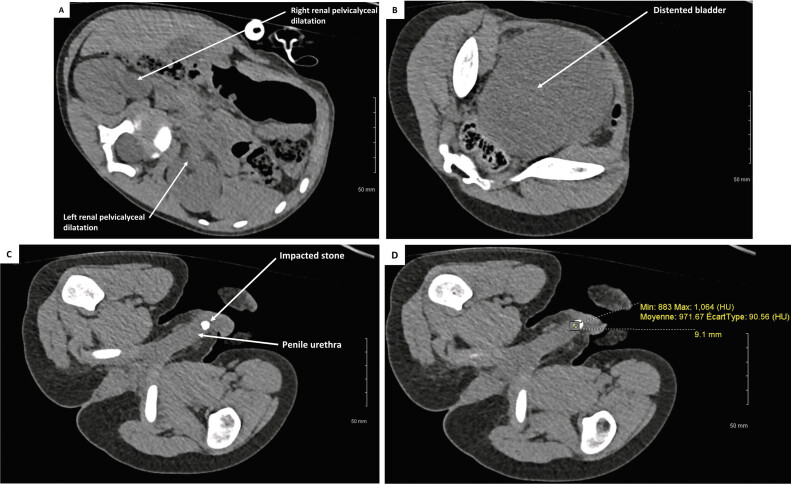
( **A** ) Bilateral hydronephrosis revealed on abdominal CT scan without intravenous contrast. ( **B** ) Significant bladder distension observed on abdominal CT scan without intravenous contrast. ( **C** ) Impacted stone located in the proximal segment of the anterior urethra on abdominal CT scan without intravenous contrast. ( **D** ) Measurement of the stone size (in mm) and density (in Hounsfield Units) by the radiologist on abdominal CT scan without intravenous contrast. CT, computed tomography.

An abdominal CT scan without intravenous contrast revealed bilateral hydronephrosis ( Fig. 1A ), urinary retention ( Fig. 1B ), and a 9-mm stone with a density of 980 Hounsfield Units that had migrated to the proximal part of the anterior urethra ( Fig. 1C, D ). Blood tests showed no evidence of acute kidney failure or electrolyte imbalance. Given the patient's significant distress and pain, and after explaining the risks and benefits of the different surgical approaches to the family, endoscopic stone extraction under general anesthesia (GA) was chosen.

First, under GA, a suprapubic puncture removed 400 mL of urine, which was sent for analysis.

Then, using a 7.5-Fr cystoscope, we identified an impacted, spiculated stone located at the base of the penile urethra, preventing any endoscopic push-back maneuver toward the bladder.

Lithotripsy was performed in the urethra using a holmium laser (VersaPulse Power Suite Holmium 20W). To minimize the risk of urethral injury, the procedure was initiated using the lowest available settings (0.5 J energy, 5 Hz frequency), with parameters gradually increased until effective fragmentation was achieved at 0.8 J and 8 Hz. These settings remained well below the upper limits typically used in adult urology (up to 15 W of power and 15 Hz of frequency), providing a substantial safety margin. In addition, laser activation was strictly limited to direct contact with the stone, and continuous irrigation was maintained throughout the procedure to reduce thermal and mechanical trauma to the urethral mucosa.

Once fragmented, the stone was pushed into the bladder for complete disintegration. The fragments were removed using a Dormia basket. At the end of the procedure, no urethral injury was observed, apart from mild inflammation. A 10-Fr urinary catheter was placed ( Video 1 ).

**Fig. 2:**
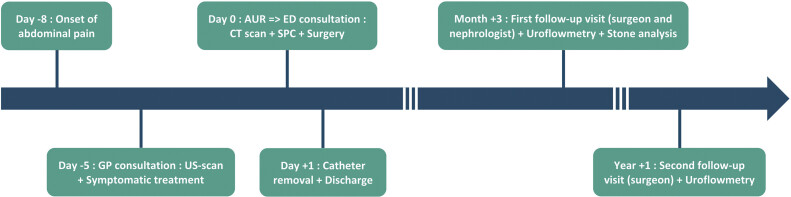
Visual timeline of the patient's clinical course. AUR, acute urinary retention; CT, computed tomography; ED, emergency department; GP, general practitioner; SPC, suprapubic catheter; US, ultrasound.

Postoperative recovery was uneventful, and the patient was discharged on the first postoperative day following catheter removal and spontaneous voiding ( Fig. 2 ).

At the 3-month follow-up visit with the pediatric surgeon and nephrologist, the patient demonstrated normal voiding without pain or dysuria, and uroflowmetry results were within normal limits. The parents expressed satisfaction with the endoscopic approach, particularly due to the rapid recovery, minimal pain, and absence of postoperative scarring. Stone analysis revealed a carbapatite calculus, likely related to a metabolic etiology (hypocitraturia). As this was the first occurrence of urological symptoms, no prior metabolic evaluation had been performed.

At the 1-year follow-up visit with the surgeon, the patient remained asymptomatic. Both the patient and their family were informed of the primary surgical risk (urethral stricture) and the importance of monitoring the urinary stream and seeking medical attention if dysuria occurred.

Regarding lithiasis, the patient will undergo annual follow-up with a nephrologist throughout childhood to monitor for recurrence of urolithiasis.

## Discussion

USI is a rare cause of AUR in children, with a very low incidence (<0.3%), 3 especially in developed countries. 4 The three main etiologies of AUR are balanoposthitis (15%), acute constipation (15%), and trauma (11.4%). 5 Due to its rarity, both the diagnosis and management of USI remain challenging in pediatric urology.

Several risk factors for USI have been identified in the literature: male sex, due to a longer and more curved urethra 6 ; urethral strictures or diverticula, which lead to urinary stasis and poor flow, thereby promoting stone formation 7 ; metabolic disorders; specific dietary regimens (including deficiencies in vitamin A, magnesium, phosphate, and vitamin B6, as well as low-protein, high-carbohydrate diets and dehydration) 4 ; and residence in endemic regions (the so-called “pediatric stone belt”) which extends from the Philippines, Thailand, and Myanmar in the Far East, through Pakistan and Iran, to the Middle East and up to Turkey, and is associated with a higher incidence of upper urinary tract calculi. 4

Clinically, symptoms often have a sudden onset, presenting as AUR or dysuria with dribbling, abdominal, flank, perineal or scrotal pain, palpable urethral mass, macro- or microscopic hematuria, nausea and vomiting, and/or urinary tract infection. 7 However, in cases of limited health care access, low parental education, or communication barriers due to young age or disability, some case reports describe a prolonged history that can lead to chronic kidney disease. 7 8

The location of the impacted urethral stone is crucial information for the surgeon to determine the most appropriate treatment approach. According to Bedii Salman, who reported the largest series of pediatric urethral calculi ( *n* = 60), 17% of the stones were in the posterior urethra, 22% in the bulbar urethra, 33% in the penile urethra, and 28% were impacted at the meatus. 9

Ultrasound is the gold standard for diagnosing suspected urolithiasis in children. 10 It is often complemented by an abdominal X-ray. Nonetheless, abdominal CT scans and ultralow-dose CT scans are still commonly used in some centers, influenced by adult-centered practices. 11 Other, less commonly used imaging modalities include kidney–ureter–bladder X-rays, cystography, and magnetic resonance imaging (MRI). 4 8 12

Despite its rarity, USI can result in significant morbidity, particularly in cases of delayed diagnosis. Both acute (electrolyte imbalances, acute kidney failure, bladder or urethral rupture, postobstructive diuresis) and chronic complications (urethral injuries including strictures and urethrocutaneous fistulas, urinary tract infection, urinary incontinence and impotence, and even rectal prolapse) 8 13 can be associated with this condition.

Following a review of the literature, no standardized guidelines currently exist for the treatment of USI in children. Management largely depends on the experience, clinical practices, and technological resources available at each medical center. Nowadays, the management of USI has progressively shifted toward endoscopic techniques, particularly in adult populations. 13

However, the use of endoscopic methods in pediatric urology remains underreported, with only 5 out of 18 studies documenting their use. This included laser lithotripsy ( *n* = 3), 7 8 14 electrohydraulic lithotripsy ( *n* = 1), 15 and endoscopic extraction ( *n* = 1), 16 with no complications reported for these endoscopic procedures.

This observation can be explained by the fact that the majority of publications reported in the literature come from developing countries ( *n* = 13/18), where access to endoscopy equipment remains limited and surgeons are sometimes less experienced in its use. The cost of a Holmium laser varies considerably, ranging from €5,000 to €60,000, depending on the brand, power, included accessories, and whether it is new or used. This high cost may partly explain the limited access to endoscopy in some developing countries. Conversely, in developed countries, endoscopy is experiencing growing adoption among pediatric urologists, 6 7 12 16 notably by building on the experience gained in adult urology, where this approach is already well-established.

The primary goal in managing AUR secondary to USI is to rapidly divert bladder urine to relieve the child and reduce pressure in the urinary tract, followed by gentle extraction of the stone, without damaging the fragile urethral mucosa, which may cause chronic disabling complications such as stricture, pain, or dysuria.

If this pathology occurs in a peripheral center without a pediatric urologist, initial management should consist of rapid urine diversion using a suprapubic catheter, followed by transfer of the patient to a tertiary care center.

As recalled by Morton et al. in their systematic review of the contemporary adult literature, the management of USI must be adapted to the size and location of the calculus, as well as the presence of associated urethral pathology. 13

Accordingly, a stone impacted at the urethral meatus or within the fossa navicularis may be gently fragmented and extracted under GA using Halstead forceps. 3

For all other USI locations along the urethra, an endoscopic “push-back” method should be attempted as a first-line approach to move the stone into the bladder, where laser fragmentation can then be performed. 8

If the “push-back” method is not feasible due to an impacted or spiked stone embedded in the urethral mucosa, laser fragmentation should be attempted directly within the urethra, as in our case.

However, in cases of large calculi (i.e., stones too large to allow the “push-back” method) and/or impacted stones (i.e., spiked calculi embedded in the urethral mucosa that prevent the “push-back” method), or when associated pathologies are present (such as diverticulum, stricture, or urethrocutaneous fistula) requiring simultaneous treatment, minimally invasive endoscopic methods should be abandoned in favor of open surgical approaches, including urethrolithotomy, urethroplasty, or cystolithotomy.

Intensive forward massage of the urethra to milk the stone should be avoided due to the risk of damaging the fragile urethral mucosa, particularly in cases of impacted or spiked calculi. 17

**Fig. 3:**
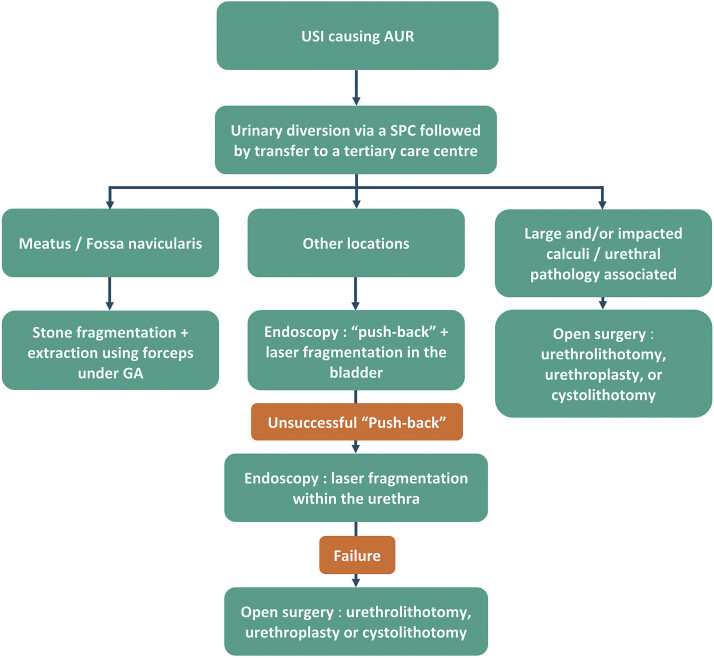
Proposed algorithm from our center for the management of urethral stone impaction. AUR, acute urinary retention; GA, general anesthesia; SPC, suprapubic catheter; USI, urethral stone impaction.

Based on this rationale, we propose an algorithm for the management of USI, as adopted in our center after our literature review ( Fig. 3 ).

The rare pediatric case reports and series on USI management using laser lithotripsy suggest good efficacy and safety. 7 8 14 However, due to the scarcity of literature on this topic and the lack of detailed reporting on laser parameters (e.g., frequency, energy), extrapolation remains challenging. Additional high-quality pediatric studies on USI management with laser lithotripsy are needed to further support our treatment rationale.

## Conclusion

AUR secondary to a USI is a challenging condition due to its rarity and the absence of standardized treatment protocols in the literature. Only a few reports exist in the pediatric literature documenting in situ laser fragmentation of urethral stones with long-term follow-up data. We propose that surgical management should be tailored to the size and location of the stone within the urethra, as well as the presence of any underlying urethral pathology. A minimally invasive endoscopic approach should be preferred whenever feasible. A systematic review of all reported cases of USI management in children, along with the establishment of international registries and consensus guidelines by professional societies, is needed to improve our understanding, treatment strategies, and long-term outcomes for this rare condition.

## Notes

Importance for a Pediatric Surgeon

- Urethral stone impaction represents a rare etiology of acute urinary retention in pediatric urology, with no standardized management guidelines currently available.
- Surgical intervention should be individualized based on stone size, location within the urethra, and the presence of any underlying urethral pathology.
- A minimally invasive endoscopic approach is preferable whenever feasible, with in situ urethral laser lithotripsy representing a safe and effective treatment option in selected cases.
